# hiHMM: Bayesian non-parametric joint inference of chromatin state maps

**DOI:** 10.1093/bioinformatics/btv117

**Published:** 2015-02-27

**Authors:** Kyung-Ah Sohn, Joshua W. K. Ho, Djordje Djordjevic, Hyun-hwan Jeong, Peter J. Park, Ju Han Kim

**Affiliations:** ^1^Department of Information and Computer Engineering, Ajou University, Suwon 443-749, South Korea, ^2^Seoul National University Biomedical Informatics (SNUBI), Division of Biomedical Informatics, Seoul National University College of Medicine, Seoul 110799, Korea, ^3^Systems Biomedical Informatics Research Center, Seoul National University, Seoul 110799, Korea, ^4^Victor Chang Cardiac Research Institute, Sydney, NSW 2010, Australia, ^5^The University of New South Wales, Sydney, NSW 2052, Australia, ^6^Center for Biomedical Informatics, Harvard Medical School, Boston, MA 02115, USA and ^7^Division of Genetics, Department of Medicine, Brigham and Women’s Hospital, Harvard Medical School, Boston, MA 02115, USA

## Abstract

**Motivation:** Genome-wide mapping of chromatin states is essential for defining regulatory elements and inferring their activities in eukaryotic genomes. A number of hidden Markov model (HMM)-based methods have been developed to infer chromatin state maps from genome-wide histone modification data for an individual genome. To perform a principled comparison of evolutionarily distant epigenomes, we must consider species-specific biases such as differences in genome size, strength of signal enrichment and co-occurrence patterns of histone modifications.

**Results:** Here, we present a new Bayesian non-parametric method called hierarchically linked infinite HMM (hiHMM) to jointly infer chromatin state maps in multiple genomes (different species, cell types and developmental stages) using genome-wide histone modification data. This flexible framework provides a new way to learn a consistent definition of chromatin states across multiple genomes, thus facilitating a direct comparison among them. We demonstrate the utility of this method using synthetic data as well as multiple modENCODE ChIP-seq datasets.

**Conclusion:** The hierarchical and Bayesian non-parametric formulation in our approach is an important extension to the current set of methodologies for comparative chromatin landscape analysis.

**Availability and implementation:** Source codes are available at https://github.com/kasohn/hiHMM. Chromatin data are available at http://encode-x.med.harvard.edu/data_sets/chromatin/.

**Contact:** peter_park@harvard.edu or juhan@snu.ac.kr

**Supplementary information:** Supplementary data are available at *Bioinformatics* online.

## 1 Introduction

Readout of genetic information in eukaryotic genomes is mediated by the dynamic chromatin environment, which regulates DNA accessibility for the gene expression machinery through chromatin compaction, associated histone modifications and incorporation of histone variants. Chromatin immunoprecipitation experiments followed by genome-wide microarray (ChIP-chip) or sequencing (ChIP-seq) have revealed that distinct genomic regulatory regions are associated with different covalent modifications of histone proteins across various organisms (Kharchenko *et al*., 2011; Liu *et al*., 2011; Mikkelsen *et al*., 2007; Park, 2009; Roudier *et al*., 2011). For example, H3K4me3 (trimethylation of histone H3 at residue lysine 4) marks active promoters, H3K4me1 marks enhancers, H3K36me3 marks transcribed gene bodies, H3K27me3 marks polycomb-repressed regions and H3K9me3 marks heterochromatin. Although there are theoretically up to $2^{n}$ possible combinations of *n* histone modifications at any given locus in the genome, in practice we only observe a small number of distinct dominant combinations, thus giving rise to the concept of chromatin states (Ernst and Kellis, 2010; Filion *et al*., 2010; Heintzman *et al*., 2009; Hon *et al*., 2008; Kharchenko *et al*., 2011; Liu *et al*., 2011; Mikkelsen *et al*., 2007; Roudier *et al*., 2011), in which each state consists of a combination of histone modifications.

A key idea underlying chromatin state analysis is to computationally identify the number and composition of chromatin states in the genome based on multiple genome-wide profiles of histone modifications and to annotate the genome with these chromatin states. These states were found to be strongly correlated with various functional genomic features such as promoters, actively transcribing gene bodies, enhancers and heterochromatins. Although many chromatin states are common across different cell types or organisms, there are indeed clear examples of cell-type-specific chromatin states consisting of unique co-occurrence of histone modifications. The H3K4me3/H3K27me3 bivalent promoter state that is prevalent in embryonic stem cells but mostly absent from terminally differentiated cells is such an example (Bernstein *et al*., 2006). Investigating co-occurrence of multiple histone marks facilitates the differentiation of more subtle features in chromatin state, such as identifying tissue-specific strong and weak enhancer regions (Ernst *et al*., 2011) and changes in co-occurrence patterns between evolutionarily distant species (Ho *et al*., 2014). Therefore, a chromatin state map is a powerful means to infer potential genome function in a systematic and automated fashion. In conjunction with transcriptomic, DNase I and transcription factor binding data, chromatin state analysis was used to infer putative biochemical functions to a large fraction of the non-coding genomic regions (ENCODE Project Consortium, 2012).

Various machine learning algorithms, such as ChromHMM (Ernst and Kellis, 2012), Segway (Hoffman *et al*., 2012), TreeHMM (Biesinger *et al*., 2013) and tiered HMM (Larson *et al*., 2013), have been developed to generate such maps to facilitate cell type-specific genome annotations in a systematic and automated fashion. All of them are based on probabilistic graphical models such as the hidden Markov model (HMM) and dynamic Bayesian network. One essential task for these algorithms is to learn the prominent combination of histone modifications. Similar to any clustering problem, it is often difficult to identify a reasonable number of combinations that can adequately capture the major variation in the data. One possibility is to estimate the adequate number of states by performing exploratory analysis such as the principal component analysis (Julienne *et al*., 2013). Another common approach is to run the HMM learning multiple times with varying state numbers and identify the best fitting model using measures such as the Bayesian Information Criterion. The inferred states do not necessarily have a one-to-one correspondence with distinct functional regions in the chromatin, but they do give a very good data-driven description of the chromatin that can act as a starting point for further bioinformatics and experimental analysis (Baker, 2011). Therefore, it is still of great interest to develop principled methods for identifying chromatin states within and across multiple genomes.

The cross-species chromatin state comparison problem was motivated by a recent model organism encyclopedia of DNA elements (modENDCODE) project that aims to systematically compare chromatin organization in *Homo sapiens* (human), *Drosophila melanogaster* (fly) and *Caenorhabditis elegans* (worm) (Ho *et al*., 2014). A naïve approach to this problem would be to compute the state map for each organism separately and then try to compare them afterward. However, this causes significant problems for interpretation because what was defined as an enhancer state in one organism is likely not identical with that from another organism. In the other extreme, we could simply concatenate the three genomes into one and infer states, but then the inferred result would be highly biased by the species with the largest genome size or by other species-specific biases in the ChIP-seq signals. Similar problems exist when comparing multiple developmental stages or cell types in the same organism. In essence, we require a method that allows the information of the state definition to be shared across multiple genomes while retaining the ability for each genome to have its own chromatin state definition.

In the context of that project, we developed a novel Bayesian non-parametric method, called hierarchically linked infinite HMM (hiHMM), to infer chromatin state maps across multiple genomes simultaneously. The application of hiHMM in the human/fly/worm cross-species comparison setting indicates that the chromatin state segmentations in individual organisms generated by hiHMM are highly comparable to the maps generated by ChromHMM (Ernst and Kellis, 2010, 2012) and Segway (Hoffman *et al*., 2012)—two widely used chromatin state segmentation algorithms (Ho *et al*., 2014). Furthermore, hiHMM is designed to address species-specific confounding factors such as variations in ChIP signal strength, genome size and co-occurrence patterns. In this article, we will present the method in detail as well as demonstrating the utility of this method using a variety of simulated and real data.

## 2 Materials and Methods

### 2.1 Statistical model for joint chromatin state inference

To address the problem of inferring consistent chromatin state definition across multiple related genomes, we employ an infinite HMM (iHMM) (Beal *et al*., 2002), a non-parametric extension of a finite state HMM, as a base model and extend it to model data from multiple conditions. For ease of model description, we consider the problem of chromatin state segmentation on *multi-species* histone modification data, in which case multiple conditions correspond to multiple species. The same statistical model can be used to describe data from different types of conditions such as multiple developmental stages or cell types.

#### 2.1.1 Background on chromatin state segmentation using HMM

We begin our model description by introducing the traditional HMM for single species data. Let $Y=(y_{1},\ldots ,y_{T})$ be an *m* × *T* matrix for histone modification data for *m* chromatin marks measured at *T* contiguous locations along the genome. Each $y_{t}={\left(y_{1t},\ldots ,y_{mt}\right)}^{T}\in R^{m}$ corresponds to the observation data at genomic location *t*. In a traditional HMM assuming *K* hidden states, each genomic location *t* is associated with a hidden chromatin state $s_{t}\in \{1,\ldots ,K\}$ from which the observation data $y_{t}$ is generated. We assume that $y_{t}$ follows a multivariate Gaussian distribution conditioned on its hidden state *s_t_* such that $y_{t}|s_{t}∼N({µ}_{s_{t}},\Sigma_{s_{t}})$ for ${µ}_{k}\in R^{m}$ and $\Sigma_{k}\in R^{m\times m}$ for k=1,…,K. The parameter ${µ}_{k}$ corresponds to the mean signal strengths from state *k* for *m* marks. The transition probabilities between hidden states are defined by the transition matrix $\pi \in R^{K\times K}$ such that $p(s_{t}|s_{t-1})=\pi_{s_{t-1},s_{t}}$. The segmentation by the chromatin states then can be naturally obtained from the hidden state sequence $S=(s_{1},\ldots ,s_{T})$. The hidden state sequence can be inferred by the Viterbi algorithm (Forney, 1973).

#### 2.1.2 Extension to hiHMM

To obtain a consistent state definition for principled comparison of chromatin states between multiple species, we propose to model the multi-species data by using an iHMM as a base model for each species data and then by coupling species-specific iHMM parameters together, so that state definition can be shared across species.

Under an iHMM (Beal *et al*., 2002; Teh *et al*., 2006), an infinite number of hidden states is assumed *a priori*, and then the number of hidden states is inferred by posterior inference from given data. An infinite dimensional transition matrix π and an infinite number of emission parameters of ${µ}_{k}$ and $\Sigma_{k}$ for k=1,2,… are defined as follows. Each row $\pi_{k}$ of the transition matrix follows the so-called Dirichlet process (DP), which defines a probability distribution on a countably infinite dimensional space of {1,2,…} (Blackwell and MacQueen, 1973; Ferguson, 1973). Formally, we have $\pi_{k}∼DP(\alpha_{0},\beta )$ where β is the base measure (‘mean’ of the DP) and α_0_ is a scale parameter controlling the concentration around the base measure. To couple each row of the transition matrix, so that the state definition can be shared across rows, a common base measure β of another DP is used, which we denote β∼GEM(γ) for a hyper-parameter γ under a stick-breaking process [for more details, refer to Teh *et al*. (2006)]. For each state *k*, the emission parameter ${µ}_{k}$ is sampled from a prior probability *H,* which we assume to be a normal distribution $N(0,\Sigma_{0}$), where $\Sigma_{0}$ is the initial covariance matrix.

In addition to the flexibility of allowing an infinite number of states a priori, an iHMM has the advantage that it naturally extends to a more general model in which multiple iHMMs can be coupled together. Suppose we have chromatin data from multiple, say *C*, species. Let c∈{1,…,C} denote the species indicator. Random variables $s_{t}^{\left(c\right)}$ and $y_{t}^{\left(c\right)}$ represent the hidden state and the observation data, respectively, at locus *t* in species *c*. We associate each species data with its own transition matrix $\pi^{\left(c\right)}$, such that each row of $\pi^{\left(c\right)}$ follows the same DP across different rows and different species. Two versions of emission parameters are considered—one that assumes a species-specific emission matrix (Model 1) and the other assuming a common emission matrix across species (Model 2). The generative model for Model 1 can be formulated as follows:

β∼GEM(γ)

$$\pi_{k}^{\left(c\right)}∼DP(\alpha_{0},\beta )$$

$$s_{t}^{\left(c\right)}|s_{t-1}^{\left(c\right)}∼\pi_{s_{t-1}^{\left(c\right)}}^{\left(c\right)}$$

(1) $${µ}_{k}^{\left(c\right)}∼H=N(0,\Sigma_{0})$$

(2) $$y_{t}^{\left(c\right)}|s_{t}^{\left(c\right)}∼N({µ}_{s_{t}^{\left(c\right)}}^{\left(c\right)},\Sigma )$$

For simplicity, we may assume $\Sigma_{0}=\sigma_{0}^{2}I$ and $\Sigma =\sigma^{2}I$ for real-valued $\sigma_{0}^{2}$ and $\sigma^{2}$ and an identity matrix I. The formulation for Model 2 is similar to the equations above except that Equations (1) and (2) are replaced by:

$${µ}_{k}∼N(0,\Sigma_{0})$$

$$y_{t}^{\left(c\right)}|s_{t}^{\left(c\right)}∼N({µ}_{s_{t}^{\left(c\right)}},\Sigma )$$

Note that the parameters are more tightly coupled in Model 2 than in Model 1 in which species-specific parameters are weakly coupled through a prior. We denote the proposed model as a hiHMM. A similar formulation that extends an iHMM to handle multi-population data was first introduced with application to the local ancestry estimation problem (Sohn *et al*., 2012) but with a different emission model and for a different data type.

#### 2.1.3 Explicit control for self-transition probability

**Fig. 1.**
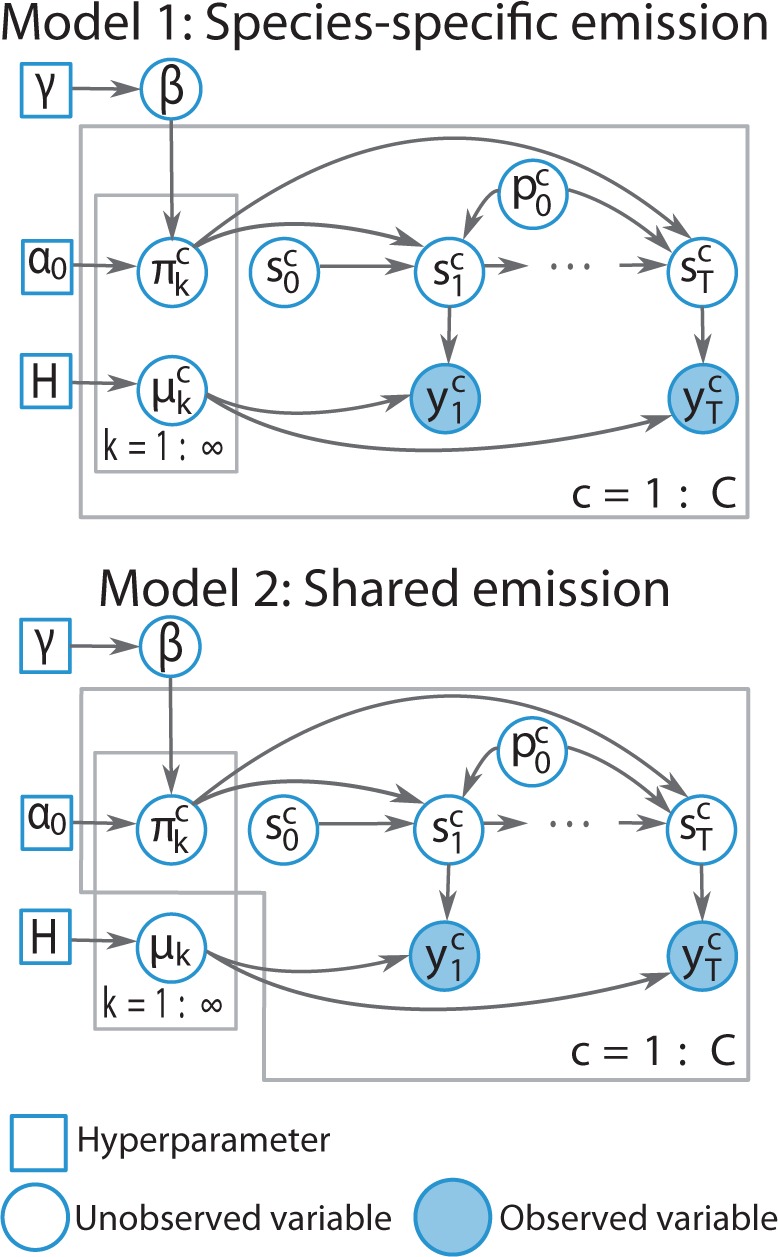
Graphical representation of hiHMM models 1 and 2

Dependencies among neighboring genomic locations may be better reflected by introducing an explicit self-transition probability. To implement this idea, we modify the transition probability as follows:

$$P(s_{t}^{\left(c\right)}=k|s_{t-1}^{\left(c\right)}=j)=p_{0}^{\left(c\right)}1(k,j)+(1-p_{0}^{\left(c\right)})\pi_{jk}^{\left(c\right)}$$

where $p_{0}^{\left(c\right)}$ denotes the self-transition probability in species *c*, and 1(k,j)=1 if *k* = *j* and 0 otherwise. We expect this model to prevent the excessive transitions between locations and to help accommodate different genome sizes and the resulting self-transition probabilities between species. The graphical representation for the final model is shown in Figure 1.

#### 2.1.4 Posterior inference

We employ a dynamic-programming technique called Beam sampling (Van Gael *et al*., 2008) for efficient posterior inference dealing with an infinite number of hidden states. It adaptively changes the number of states over iterations, so at each iteration, it tries to increase the state space, and based on the new parameters with increased number of states, the hidden state sequence is sampled. Then we check whether there is a hidden state having no association. We remove those states from our state space. On the basis of this new hidden state sequence, we re-sample HMM parameters. We repeat this until *L_i_*, the mean of the last *m* posterior samples at *i*th iteration, converges. That is, the convergence criteria is $|L_{i}-L_{i-1}|/|L_{i-1}|<1.0\times 10^{-5}$. The other iHMM parameters and the self-transition probabilities are inferred by Gibbs sampling.

#### 2.1.5 Parameter initialization

Like most HMM-based learning methods, using good initial parameters is important in obtaining good learning outcome. To obtain reasonable initial emission and transition matrices, we first concatenate all genomes together and perform a *k*-mean clustering step. This also encourages coupling of emission matrices in different species in case of Model 1.

### 2.2 Simulations

#### 2.2.1 Simulation scenarios

We first demonstrate the main benefit of the proposed models on simulated datasets. The simulation scenarios are mainly motivated by a recent modENCODE project that compares the chromatin organization in human, fly and worm to reveal common and species-specific chromatin states (Ho *et al*., 2014). We simulated histone modification data with *M* = 8 marks from *C* = 3 species (Supplementary Fig. S1). The transition matrix and the emission parameters are simulated under various scenarios below. Non-zero elements in the emission matrix correspond to the enriched marks. At each parameter setting, a hidden state sequence and the corresponding observation sequence of length *T* is generated under the standard HMM model per species, and this is repeated 50 times.

We describe the default simulation setting for the HMM parameters first. For each state *k* among the assumed *K* hidden states, one or two histone marks are randomly chosen to have non-zero signal for all the species. The average signal strength *s_km_* of each enriched mark *m* in state *k* is randomly sampled from uniform distribution *U*(1, 4) for k=1,…,K−1 and from U(0.05,1) for *k* = *K* to simulate a chromatin state with low signal. As the dynamic range of ChIP-seq signal may not be identical across species due to technical differences, we further incorporate a discrepancy parameter ρ, such that the signal strength for the mark in species *c* is defined as ${µ}_{km}^{\left(c\right)}=s_{km}$ if *c* = 1 and ${µ}_{km}^{\left(c\right)}=s_{km}(1+{\left(-1\right)}^{\left(c-1\right)}\rho )$ for c≥2. We keep ρ relatively small compared with the average signal strength in *U*(1, 4); ρ=0.1 in most scenarios below except for one scenario to examine the performance behavior with respect to ρ. Finally, we set one state as species specific by making one of the enriched marks at the state as un-enriched (i.e. signal strength of zero) for species c≥2. The transition parameter is defined as $\pi^{\left(c\right)}=p_{0}^{\left(c\right)}I_{K}+(1-p_{0}^{\left(c\right)})\pi_{0}^{\left(c\right)}$, where $\pi_{0}^{\left(c\right)}=\pi_{0}+\pi_{c}$, $\pi_{0}$ is a random sparse matrix with 30% of randomly uniformly distributed non-zero elements and constant across species and $\pi_{c}$ is a random sparse matrix with 10% of non-zero elements, which are species-specifically sampled. We normalize each row of $\pi^{\left(c\right)}$ to have row-sum of one. $I_{K}$ is an identity matrix of size *K* × *K*. We fix *K* at 10.

**Scenario I**

We examine the effect of different genome size. The following three settings are considered in which either the genome size *T* or the self-transition probability *p*_0_ varies between species. We fix ρ at 0.1.

- Scenario I-1: The same genome size of T=(2000,2000,2000), for each of the three species, and the same average self-transition probability $p_{0}=(0.9,0.9,0.9)$.
- Scenario I-2: Different genome size of T=(2000,5000,10000) and the same self-transition probability $p_{0}=(0.9,0.9,0.9)$.
- Scenario I-3: Different genome size T=(2000,5000,10000) and different self-transition probability $p_{0}=(0.7,0.8,0.9)$.

**Scenario II**

We study the effect of different ChIP-seq signal strength across species. We vary ρ from 0.1 to 0.5 by step size of 0.2. The genome size and the self-transition probability were the same as those in scenario I-1 in which the genome size and the self-transition probabilities are the same across species.

**Scenario III**

The effect of the number of species-specific states is studied in this scenario. We vary the number of species-specific states *n* from 0 to 2. The other parameters were the same as in scenario I-1.

#### 2.2.2 Performance comparison

We compare the hiHMM Model 1 (hiHMM1) and Model 2 (hiHMM2) with an iHMM combined with the self-transition model introduced in section 2.1.3 (iHMM+p0) that assumes a common emission matrix and a common transition matrix for all species, the iHMM without such additional self-transition model (iHMM), so that transition probabilities are defined as $P(s_{t}^{\left(c\right)}=k|s_{t-1}^{\left(c\right)}=j)=\pi_{jk}^{\left(c\right)}$ and the existing methods of ChromHMM and Segway. In hiHMM 1 and hiHMM2, we set the initial number of states *K*_0_ as 7, the variance parameters of $\sigma^{2}=1$ and $\sigma_{0}^{2}=1$. The traditional HMMs with *K* = 10, 13, 16 states are also compared for which a Matlab toolbox for HMM written by *K*. Murphy was used in its default setting (http://www.cs.ubc.ca/murphyk/Software/HMM/hmm.html) except that the full covariance matrix is assumed. In all cases other than hiHMM1 and hiHMM2, genomes from different species were concatenated as one sequence and used as input.

The segmentation performance is compared in two main aspects: the accuracy of clustering genomic loci by hidden state labels and the segment boundary detection accuracy. We use the adjusted Rand Index (RI) for the former, and the *F*-measure computed from precision and recall for the latter. RI (Rand, 1971) is a traditional measure for clustering accuracy and considers the number of pairs of samples whose labels are correctly assigned, that is the number of pairs that are in the same (or different) cluster (i.e. the same hidden state) both under the ground truth and under the estimated labels. The adjusted RI is the normalized difference of the RI and its expected value under the null hypothesis, so that the expected value of two random partitions becomes zero (Hubert and Arabie, 1985).

For segment boundary detection accuracy, precision is defined as the number of true boundaries among detected boundaries divided by the total number of detected boundaries, and recall is the number of true boundaries among detected boundaries divided by the total number of true boundaries. *F* measure is the harmonic mean of precision and recall, that is, F=2(precision·recall)/(precision+recall).

### 2.3 Real data applications

#### 2.3.1 Running hiHMM on fly and worm ChIP-seq data

The fly (genome assembly version dm3) and worm (genome assembly version WS220) ChIP-seq and RNA-seq data were generated by modENCODE consortia. All input-normalized ChIP-seq signal tracks were downloaded from the ENCODE-X interactive faceted browser: http://encode-x.med.harvard.edu/data_sets/chromatin/. The original fly and worm ChIP-seq data were in 10-bp resolution. All tracks were re-binned to 100 bp resolution by taking the mean of 10 consecutive bins. Data from multiple histone modifications were concatenated as columns into a tab-delimited format. Bins that overlapped unmappable regions were removed (mappability regions were downloaded from https://www.encodeproject.org/comparative/chromatin/#mappability).

hiHMM was run in Matlab with default parameters: 200 burn-in iterations, which means the first 200 samples, are discarded during iterations for posterior inference and then 10 consecutive posterior samples are collected to produce the final Maximum-A-Posterior output. For each comparison, all available histone modification profiles produced by ChIP-seq experiments that are common across the targeted species and cell types were used (Supplementary Table S1). Chromatin states were trained on representative fly chromosomes 2 L, 2LHet, X and XHet and worm chromosomes II, III and X, as per the modENCODE study (Ho *et al*., 2014). Our prior experience suggests that training with all or only this representative subset of chromosomes in these organisms make very little difference in terms of the resulting chromatin state definition. Nonetheless, the hiHMM program is scalable to analyze all the chromosomes—which would be useful for exploring any previously uncharacterized chromatin landscapes.

Emission matrices from hiHMM output were examined and states were named based on chromatin state definitions in previous studies as well as overlap with expressed or unexpressed genes (Ernst *et al*., 2011; Kharchenko *et al*., 2011) (Supplementary Figs. S2 and S3). A custom R script is used to rename the states and re-introduce unmappable regions as State 0.

#### 2.3.2 Chromatin state statistics

Genomic coverage was calculated as the percentage of the mappable genome that is occupied by each state, at the bin level. Expression odds ratio was calculated as the ratio of the number of expressed versus silent genes that overlapped with each chromatin state, divided by the genome-wide ratio of the number of expressed versus silent genes. A gene was considered expressed if its mRNA expression levels were >1 RPKM (Reads Per Kilobase per Million mapped reads). Gene body overlap was calculated as the percentage of bins annotated to each chromatin state that occur between the transcription start site (TSS) and transcription end site (TES) of an annotated gene.

#### 2.3.3 Meta-gene chromatin state enrichment profile

A meta-gene matrix was constructed from all annotated protein coding genes that were at least 1300 bp in length and do not overlap another gene within 500 bp of its TSS or TES. Protein-coding gene annotation was downloaded from https://www.encodeproject.org/comparative/transcriptome/. We further excluded genes that occurred within 1000 bp of a chromosome start or end. The meta-gene matrix contains the chromatin state annotations of each ‘representative’ gene extending to 500 bp upstream of the TSS and 500 bp downstream of the TES. Enrichment profiles are presented as heatmaps where the color indicates the percentage of genes that have been annotated with that particular chromatin state at that relative genomic position. Meta-gene profiles of expressed and silent genes were computed separately.

#### 2.3.4 Inter-sample chromatin state co-occurrence

Fold change was calculated as the observed number of bins that transitioned between any two chromatin state annotations divided by the expected number. The expected number was the mean number of bin transitions between those two states in 1000 Monte Carlo simulations with a randomized chromatin state assignment, preserving the relative genomic coverage of each state. Fold change was truncated between one and five for simplified visualization and interpretation.

#### 2.3.5 Co-occurrence matrices

Co-occurrence of genomic chromatin state annotation between experiments was calculated as the number of bins that were annotated as a particular chromatin state combination in two experiments divided by the total number of bins annotated to those states in the respective experiments. This gives a value between 0 and 1, which is presented in a heatmap.

#### 2.3.6 Gene ontology enrichment of target genes in a region

Official gene symbols for all genes that overlapped with the selected regions were submitted to the DAVID bioinformatics tool (Huang *et al*., 2009). The Benjamini adjusted *P* value (Benjamini and Hochberg, 1995) of the 10 most significant gene ontology (GO) biological process results are presented for each analysis.

## 3 Result

### 3.1 Simulation study

#### 3.1.1. An illustrative example

**Fig. 2.**
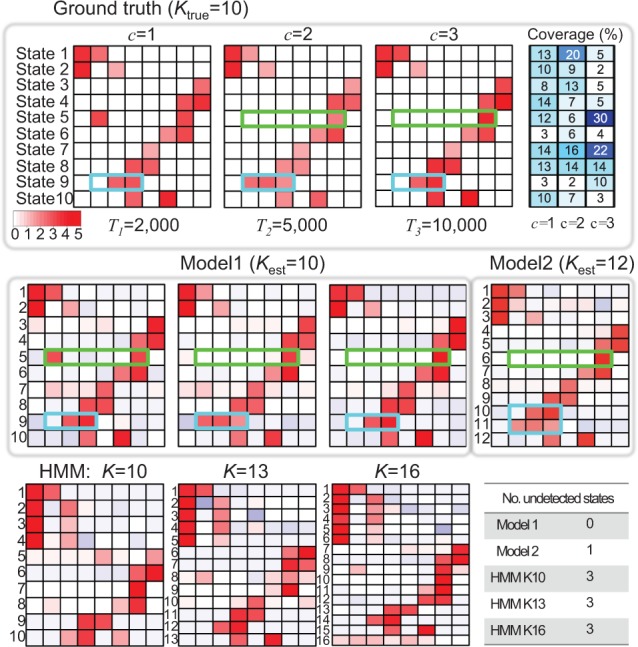
An illustrative example using a toy simulated dataset. The heatmaps show the emission parameters of the ground truth with *K* = 10 chromatin states in three species (top panel) and the hiHMM and HMM estimated parameters. As State 5 (green box) and State 9 (cyan box) have different combinations of enriched marks between species, the number of distinct chromatin states across all the species is 12. Model 1 recovers the correct number of states and the enriched marks. Model 2 recovered one of the two species-specific state but missed the species-1-specific State 5. The standard HMMs miss three states even when a large *K* is assumed

We compare the true simulated emission matrices and the estimated ones by each algorithm on an example dataset with *K* = 10 chromatin states model (Fig. 2). The true model contains two species-specific states (State 5 and State 9) that have species-specific mark combinations. Therefore, the actual number of distinct chromatin states across all the species can be viewed as 12. The genome size *T* was 2000, 5000 and 10 000 for each of the three species.

As shown in Figure 2, Model 1 recovers the correct number of hidden states and the correct mark combination. Model 2 recovers one species-specific state correctly (State 9 in a cyan box Fig. 2), but it misses the mark combination of the species-1-specific State 5 (green box in Fig. 2), possibly due to the shorter genome length of species 1, resulting in lower overall representation of the state during joint learning. Instead, two states corresponding to State 1 are recovered with slightly different signal strengths. This appears to happen because the average signal strength is not identical between species. In contrast, Model 1 correctly recovers all the states as it allows species-specific emission matrix that can have different signal strength.

The standard HMMs fail to recover species-specific states even when a large *K* of 16 was assumed. For example, although the signal of the second mark in State 5 for species 1 is relatively strong, standard HMMs either miss the state completely (the case of *K* = 10) or the recovered signal is very weak (cases of *K* = 13, 16). Again, this seems to be because of the shorter genome size of species 1 and its relatively low coverage. Moreover, State 9 in species 2 is completely missing in all cases of *K* = 10, 13, 16 possibly because it is specific to species 2 and its coverage is very low (1.7%). In summary, a standard HMM fails to recover the correct states, likely due to the existence of species-specific states, different genome size and possibly different ChIP-seq signal range.

#### 3.1.2 Comparison of segmentation accuracy

**Fig. 3.**
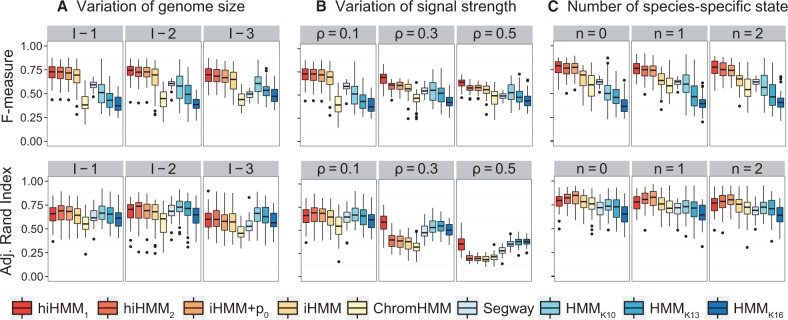
Performance comparison on simulated datasets based on three aspects: (**A**) the effect of different genome size (scenario I), (**B**) the effect of different ChIP-seq signal strength between species (scenario II) and (**C**) the effect of the number of species-specific states (scenario III). The plots in the top row show the *F*-measure for the segment boundary detection, and the plots in the bottom row show the clustering accuracy by segment labels in terms of adjusted RI. Note that iHMM+p0 and iHMM correspond to the plain iHMM combined with or without the self-transition model introduced in Section 2.1.3, respectively

The overall performance over 50 simulated datasets per parameter setting across scenarios is compared in terms of the adjusted RI between the true state labels and the estimated ones, and the segment boundary detection accuracy of *F*-measure. Figure 3 shows boxplots for the accuracy of each algorithm (Fig. 3A: scenario I, B: scenario II, C: scenario III).

Both Model 1 (hiHMM1) and Model 2 (hiHMM2) perform superior or comparable to the other algorithms across all the scenarios. HMM with *K *= 13 or 16 in which the assumed number of states is larger than the true number of states consistently perform the worst especially in terms of *F*-measure for the boundary detection accuracy. The traditional HMMs and ChromHMM tend to over-segment the genome and produce a large number of false-positive segment boundaries, resulting in low precision. When comparing hiHMM1 and hiHMM2, their performances are comparable overall in both aspects. When the signal strength variation ρ is low, hiHMM2 outperforms hiHMM1 in terms of clustering accuracy (e.g. *P* value of 0.02 from a paired *t*-test in case of scenario II-1), but as the discrepancy increases, hiHMM1 significantly outperforms hiHMM2 with a *P* value < $2.2\times 10^{16}$ (Fig. 3B). We find that the performance change of each algorithm is the most dominant in scenario II for which the signal strength variation ρ is changed.

Regarding computation time, hiHMM1 or hiHMM2 each took less than 2 min on average per simulation dataset (scenario I-1) in a single Intel(R) Xeon(R) CPU E5-2650 (8-core, 2.00 GHz) with 32 GB RAM (DDR3, 1600 MHz) and CentOS 6.4 (64-bit architecture), which is shorter than that from Segway (around 3 ∼ 4 min) but longer than that from ChromHMM (<1 min).

### 3.2 Real data analysis

#### 3.2.1 Case study 1: hiHMM identifies species-specific chromatin states in fly and worm

The analogous developmental stage of stage 3 larva (L3) in fly and worm was selected for this cross-species chromatin state analysis. hiHMM was run using 25 starting states with Models 1 and 2 on the combined data and 30 starting states using Model 2 to capture more species-specific states.

**Fig. 4.**
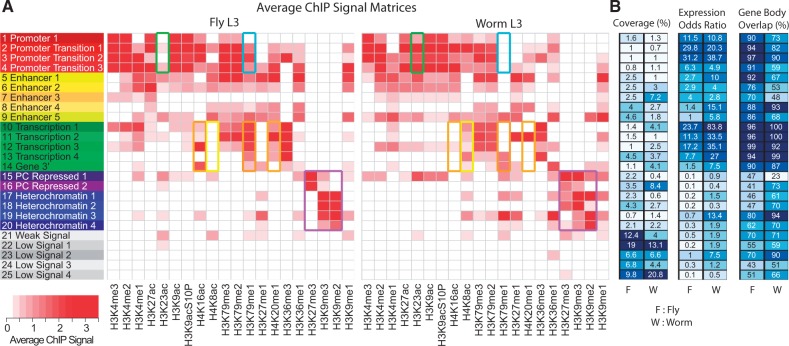
Cross-species chromatin state analysis. (**A**) ChIP signal matrix showing the average observed histone modification profiles for each of 25 states inferred by the hiHMM algorithm (Model 1) in fly and worm. Species-specific differences are highlighted. (**B**) Percentage of genome covered by the state (coverage), relative enrichment of expressed genes per state (expression odds ratio) and the percentage of state annotations that occur between the TSS and TES of annotated genes (gene body overlap)

Chromatin state analysis of fly L3 versus worm L3 shows both shared and unique patterns of chromatin mark co-occurrence between the two species (Fig. 4A). We found 25 states that grouped into six categories: promoters, enhancers, gene body, heterochromatin, repressed and low signal. Most states are conserved and have similar compositions but with some clear differences. Fly promoter states (red) show a distinct lack of H3K23ac when compared with worm (green highlight in Fig. 4A). Conversely, worm promoter states lack H3K79me1 when compared with fly (BLACK highlight in Fig. 4A). Genic and transcription states (green) in fly show enrichment of H4K16ac, H3K79me1 and H4K20me1, all of which are largely absent in the same states in worm (orange highlight in Fig. 4A). H4K8ac, on the other hand, is enriched in these states in worm but completely absent in fly (yellow highlight in Fig. 4A). Further differences are visible in the repressed (purple) and heterochromatin (black) states. In fly, there is a clear differentiation of repressive histone modifications between the two state classes, whereas the marks consistently co-occur in worm (purple highlight in Fig. 4A). To verify that these differences were representative of the data and not simply training errors, we examined the data in a genome browser and visually confirmed the differences (Supplementary Figs. S4–S6).

To compare the performance of hiHMM Model 1s and 2, we also learned a shared chromatin state definition using Model 2 (Supplementary Fig. S7). Increasing the number of states from 25 to 30 allowed for the identification of a greater number of species-specific states, apparent from an absence of the state (0 genomic coverage) in one species (Supplementary Fig. S8). When examining the state co-occurrence matrix between the two models, there are obvious anomalies in species-specific states 10 and 12 (Supplementary Fig. S9).

#### 3.2.2 Case study 2: hiHMM identifies developmental stage-specific loci in fly

Three fly developmental stages were chosen for chromatin state comparison: late embryo (EL), third instar larvae (L3) and adult head (AH). hiHMM was run on the combined datasets using 25 starting states with Models 1 and 2.

Jointly learned average ChIP signal matrices for three developmental stages in fly show that the majority of observed histone modification combinations and their genomic occurrence remain stable during development (Supplementary Figs. S10 and S11). H3K79me1, however, shows a marked reduction in enrichment in active states in AH compared with EL and L3 stages (Supplementary Fig. S12). Although this difference is interesting, it is secondary to our ensuing analysis of differential state co-occurrence during development. Examining the co-occurrence between Models 1 and 2 shows a very high level of concordance, so we continue the analysis using the shared chromatin state definition learned by Model 2 (Supplementary Fig. S13).

**Fig. 5.**
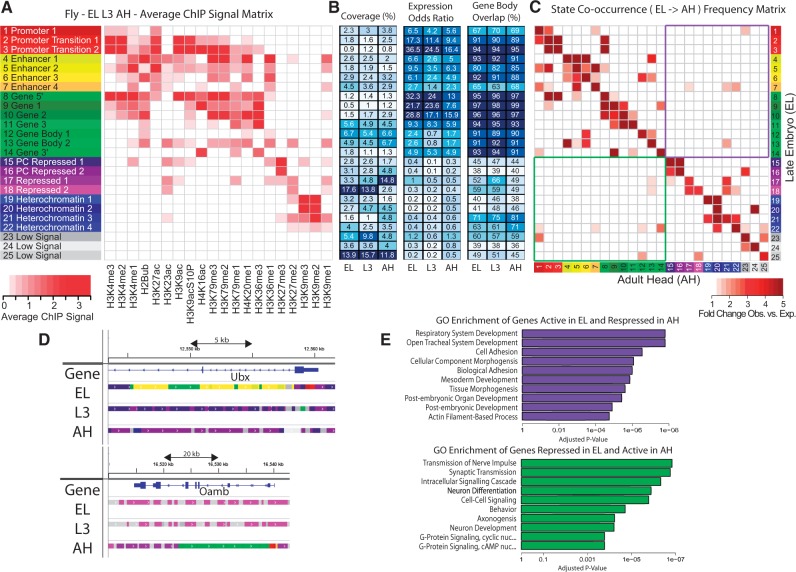
Chromatin state characterization and analysis across developmental stages in fly. (**A**) ChIP signal matrix showing the average observed histone modification profiles for each of 25 states jointly inferred by the hiHMM algorithm (Model 2) for three stages of fly development: late embryo (EL), stage 3 larvae (L3) and adult head (AH). (**B**) Percentage of genome covered by the state (coverage), relative enrichment of expressed genes per state (expression odds ratio) and the percentage of state annotations that occur between the TSS and TES of annotated genes (gene body overlap). (**C**) Chromatin state co-occurrence between two developmental stages in fly (EL and AH). The observed versus expected fold change of the co-occurrence of each state in EL and each state in AH is shown. On the basis of this analysis, we selected significantly over represented co-occurrence regions to investigate and characterize the genes involved through gene set enrichment analyses. (**D**) Genome browser views of representative genes Ubx and Oamb with three stage chromatin states. These genes were identified through chromatin state co-occurrence analysis as having different chromatin states in EL and AH. (**E**) The top 10 GO biological processes enriched in the genes that are within regions of the genome that changed from an active state in EL to a repressive state it AH (top panel) or vice versa (bottom panel)

Using these chromatin state maps, we implemented an unbiased approach for identifying developmentally regulated genes from chromatin state co-occurrence between two developmental stages—EL and AH—in fly (Fig. 5). In total, 1659 genes from regions that transitioned from an active (promoter, enhancer, gene) state in EL to an inactive (repressed, heterochromatin, low signal) state in AH (Fig. 5C, top right) were strongly enriched for multiple developmental GO terms, including ‘Respiratory system development’ (*P* value 1.66 × 10^−^^8^) (Fig. 5D). Similarly, 1889 genes that changed from inactive in EL to active in AH (Fig. 5C, bottom left) were strongly enriched for terms expected from a fully developed organism, including ‘Transmission of nerve impulse’ (*P* value 1.48 × 10^−^^7^). These transitions in developmental regulation are clearly visible in a genome browser when the chromatin state tracks are visualized (Fig. 5E).

## 4 Discussion

The main innovation of our approach is that it provides a flexible framework to enable information sharing between multiple HMMs. The main difficulty of joint analysis of related datasets is how to obtain a consistent state definition or the mapping between a set of states defined in one species and the one in another species. If one were to apply iHMM separately on each of the datasets, we face the problem of mapping state definitions between species. The proposed hiHMM solves this problem by assuming a common state space definition across all the datasets. It is one of the main advantages of using the non-parametric model based on the so-called hierarchical DP, which is the core component of iHMM. Under a hierarchical DP prior for data from multiple groups, each group is associated with a group-specific DP and then those DPs share a common base measure that is another DP. This is the major mechanism how the atoms from each DP can be shared across groups. Model 1 couples the state definition more loosely than Model 2 since in Model 1 information is shared via the prior only, whereas Model 2 explicitly employs a shared emission matrix.

One distinguishing feature of hiHMM is that we can jointly infer emission values for genomes of differing lengths without the dominating influence of a longer genome on the result or the need to perform compensatory subsampling during training. This is a major limitation of existing methods that concatenate multiple samples for joint learning that is overcome using hiHMM.

Similar to Segway or the HMM approach of Kharchenko *et al*. (2011), hiHMM directly models continuous ChIP signal values and therefore alleviate the need of selecting a binary threshold cutoff. We observe that this feature is potentially important for differentiating within a class of similar states, highlighted by the interesting and diverse characteristics we observe in low signal states (Supplementary Fig. S4).

Both our simulated and real data analyses show that the advantages of Model 1 become more evident when the data discrepancy across different conditions is large, for example, in the case of multiple species data. In terms of revealing interesting biology, the ability to infer sample-specific emission matrix parameters (as in Model 1) allows for intuitive and detailed comparison of chromatin mark combinations between different species or cell types. This is evident from our 25 state analyses in fly versus worm in case study 1. In addition to the finding that fly and worm have different chromatin modifications compositions in heterochromatin, here we observed several previously unreported differences between the two species as described above, most notably the relative depletion of H3K23ac and enrichment of H3K79me1 in fly promoter states, as well as multiple differences in the transcription states. Model 1 was also useful in identifying the unexpected changes in the distribution of H3K79me1 during fly development. Many of these observations were not possible in previous studies that did not include these marks in their comparison (Supplementary Fig. S14), and they may suggest different mechanisms of genetic regulation between the two species.

Jointly learned and shared emission matrix parameters (as generated by Model 2) provide an easily interpretable platform on which to compare multiple samples from the same genome without the confounding factor of different state definitions. Additionally, Model 2 enjoys better statistical properties such as faster convergence and shorter running time, so Model 2 would be a better choice when the discrepancy between genomes is expected to be relatively small as in the case of different developmental stages or environmental conditions in a single species.

By applying hiHMM to this newly compiled dataset, we were able to identify two previously uncharacterized chromatin states that we have named ‘Repressed’ states in the fly development analysis (states 17 and 18 in Fig. 4A). These two states combined constitute roughly 18% of the fly genome and are almost exclusively characterized by marks that were not profiled in previous studies, and so these states were missed.

Although hiHMM allows learning the optimal number of states from the data in principle, the likelihood surface of the model over the used parameter space is quite flat. Therefore, similar posterior probability value can be obtained through different parameter value combinations, and this makes the recovery of the correct number of states still challenging. For systematic analysis of this issue, we examined the effect on the accuracy and the number of inferred states of three hyper-parameters: the initial number of states *K*_0_ and the two variance parameters: $\sigma_{0}^{2}$ in the prior distribution for the emission matrix and $\sigma^{2}$ for generating the observation signal given the hidden state and the emission matrix (Supplementary Figs. S15 and S16 for Model 1 and Model 2, respectively). The smaller $\sigma^{2}$ produced better segmentation accuracy, which is expected as the variance in the emission model should be small enough to assign the observation signal to the correct state. Assuming proper normalization by variance, $\sigma_{2}=1$ seems to produce the best result. The number of inferred states depended on all the three parameters but most significantly by $\sigma_{0}^{2}$. We see that $\sigma_{0}^{2}$ should be large enough to compactly capture the varying signals from enriched marks as the prior mean for the emission matrix is zero. For large $\sigma_{0}^{2}$ (>2), the number of inferred states becomes less affected by other parameters. The deviation from the initial number of states to the inferred number is also mostly determined by $\sigma_{0}^{2}$ but not significantly by others. It appears to be a good practice to set $\sigma_{0}^{2}$ to be around the mean signal strength of the enriched marks. The initial number of states should be selected close to the true number of states. Automatic estimation of these hyper-parameters would need further investigation in our future work.

Another important issue is to get a reasonably good initial state assignment, which encourages consistent state-definition across species. This issue is especially critical with hiHMM1 in which the species-specific models are loosely connected. Our current joint initialization scheme of concatenating different genomes as one and applying *k*-means clustering works reasonably well, but this would be investigated further in our future study.

In this article, we demonstrated a variety of features of hiHMM that makes it useful for cross-sample joint chromatin state inference. We have devised two models of hiHMM, each having advantages and limitations of interpretation and inference. The flexibility of using both learning models allows for a more comprehensive analysis during different applications and experimental designs.

## Supplementary Material

Supplementary Data
