# Supplementary material for: hiHMM: Bayesian non-parametric joint inference of chromatin state maps
Source: Bioinformatics. 2015 Feb 27;31(13):2066–74. doi: 10.1093/bioinformatics/btv117 (PMC4481846; doi:10.1093/bioinformatics/btv117)
Supplement: Supplementary Data [file supp_31_13_2066__index.html]

hiHMM: Bayesian non-parametric joint inference of chromatin state maps — hiHMM: Bayesian non-parametric joint inference of chromatin state maps — Supplementary Data 

# hiHMM: Bayesian non-parametric joint inference of chromatin state maps

## Supplementary Data

files

**Files in this Data Supplement:**

- Supplementary Data - docx file
