# Supplementary material for: hiHMM: Bayesian non-parametric joint inference of chromatin state maps
Source: Bioinformatics. 2015 Feb 27;31(13):2066–74. doi: 10.1093/bioinformatics/btv117 (PMC4481846; doi:10.1093/bioinformatics/btv117)

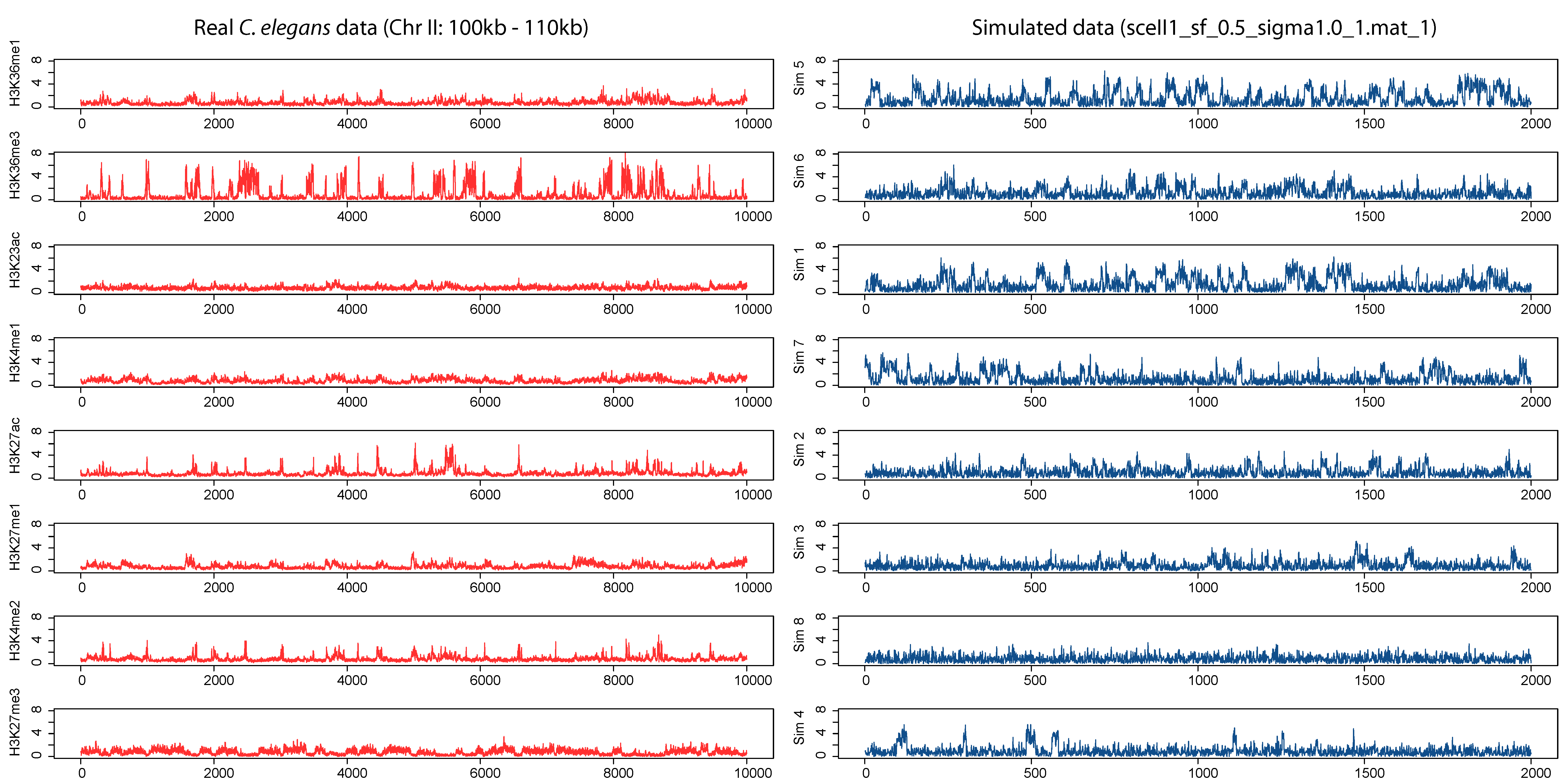


**Fig S1. *Comparison between simulated data and real data.*** We plot the values of our simulated data and real data to show that our simulated data contains realistic features including sharp peaks, broad domains and background noise.


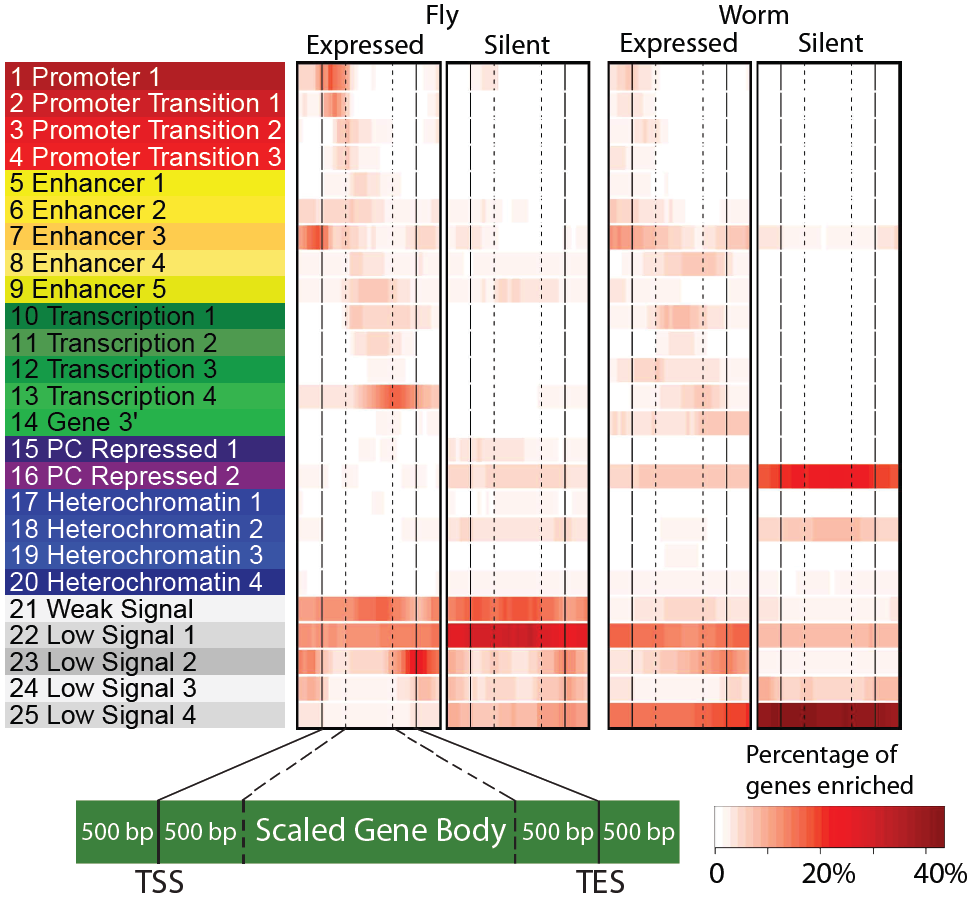


**Fig S2. *Chromatin State Meta Gene Enrichment Profiles - Fly vs Worm - Model 1.***  Heatmaps showing the spatial enrichment of each chromatin state in relation to the average `meta gene’.


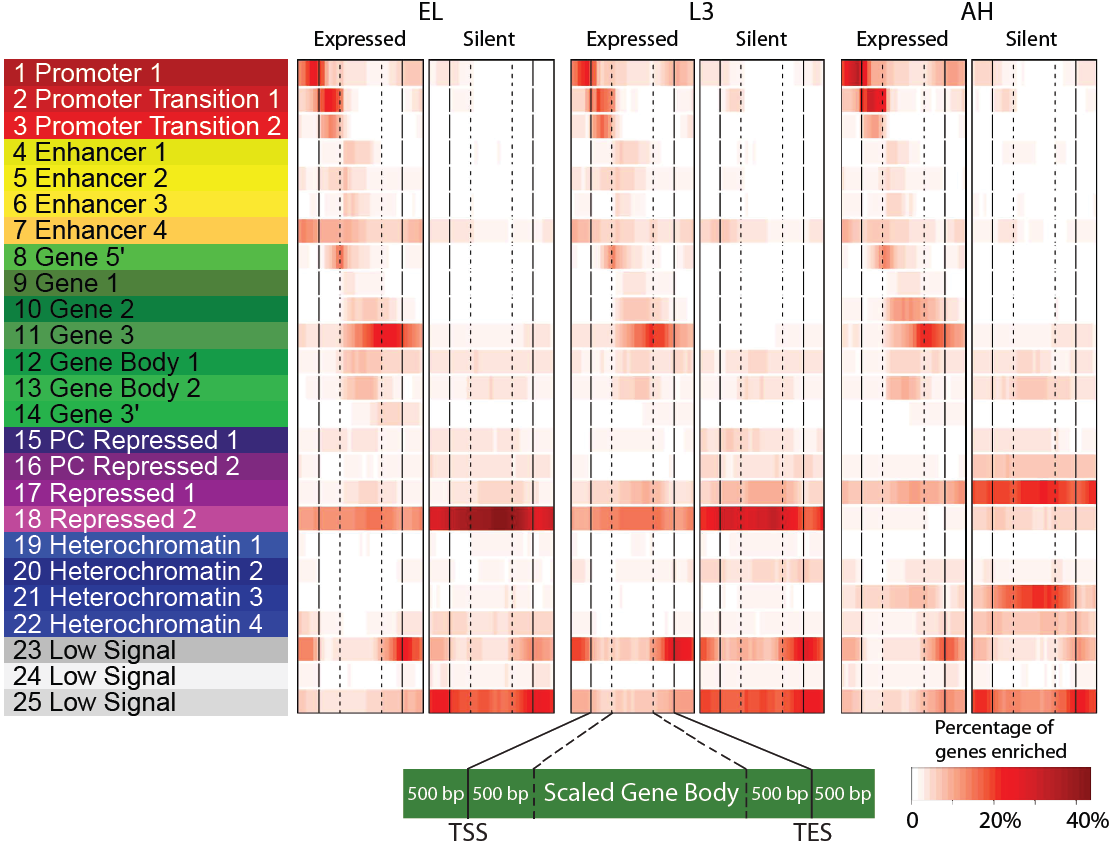


**Fig S3. *Chromatin State Meta Gene Enrichment Profiles - Fly 3 Stages - Model 2.*** Heatmaps showing the spatial enrichment of each chromatin state in relation to the average `meta gene’.

**
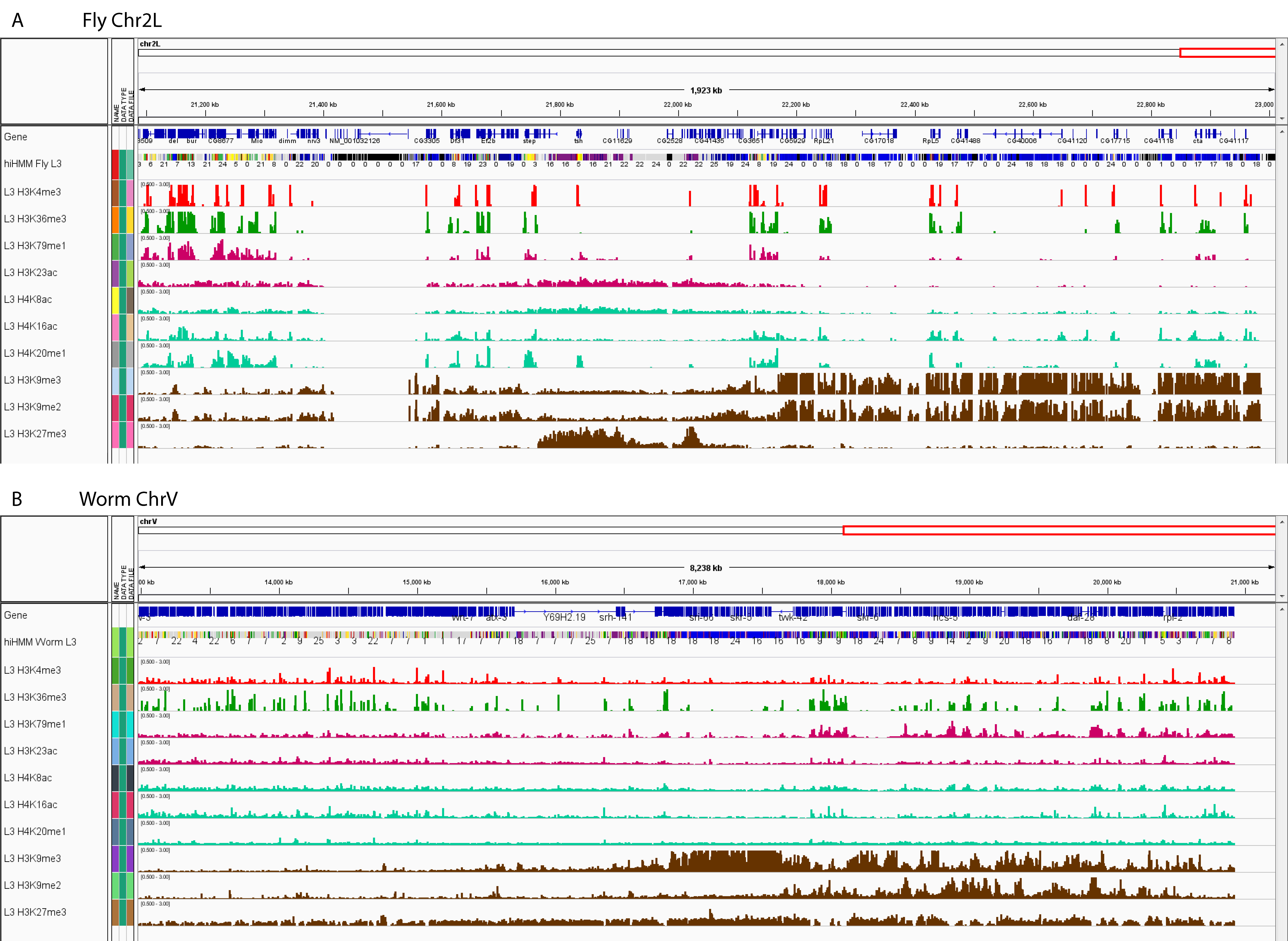
**

**Fig S4. *IGV browser plots showing differences in repressive state composition between fly and worm.*** H3K9me3, H3K9me2 and H3K27me3 marks appear in dark brown colour, top to bottom respectively. Repressive states are coloured blue and purple in the hiHMM chromatin state track (below gene annotations).

**
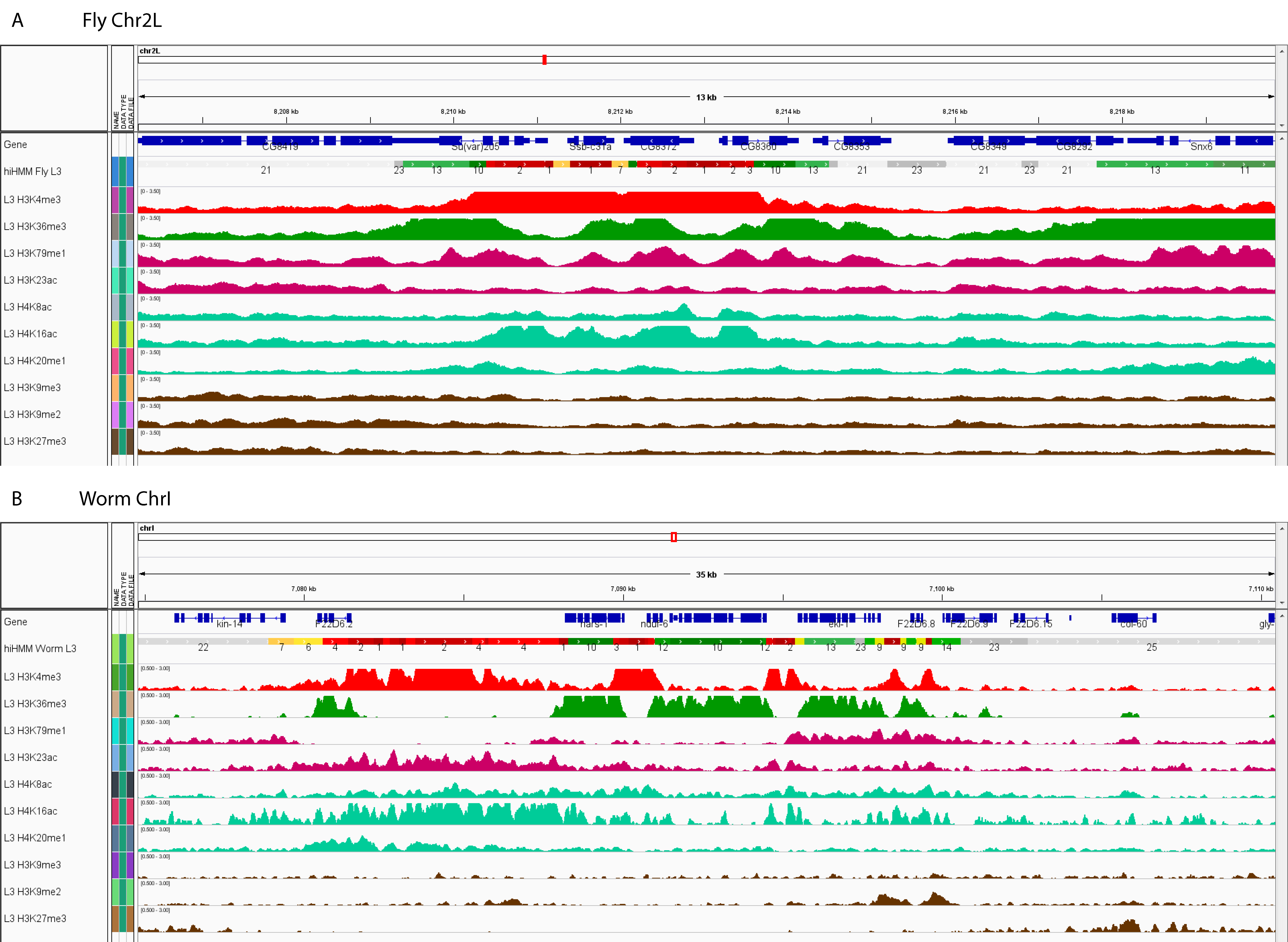
**

**Fig S5. *IGV browser plots showing differences in promoter state composition between fly and worm.*** H3K79me1 and H3K23ac are coloured in purple. Promoter states are coloured red in the hiHMM chromatin state track (below gene annotations) and marked by H3K4me3 (red signal).

**
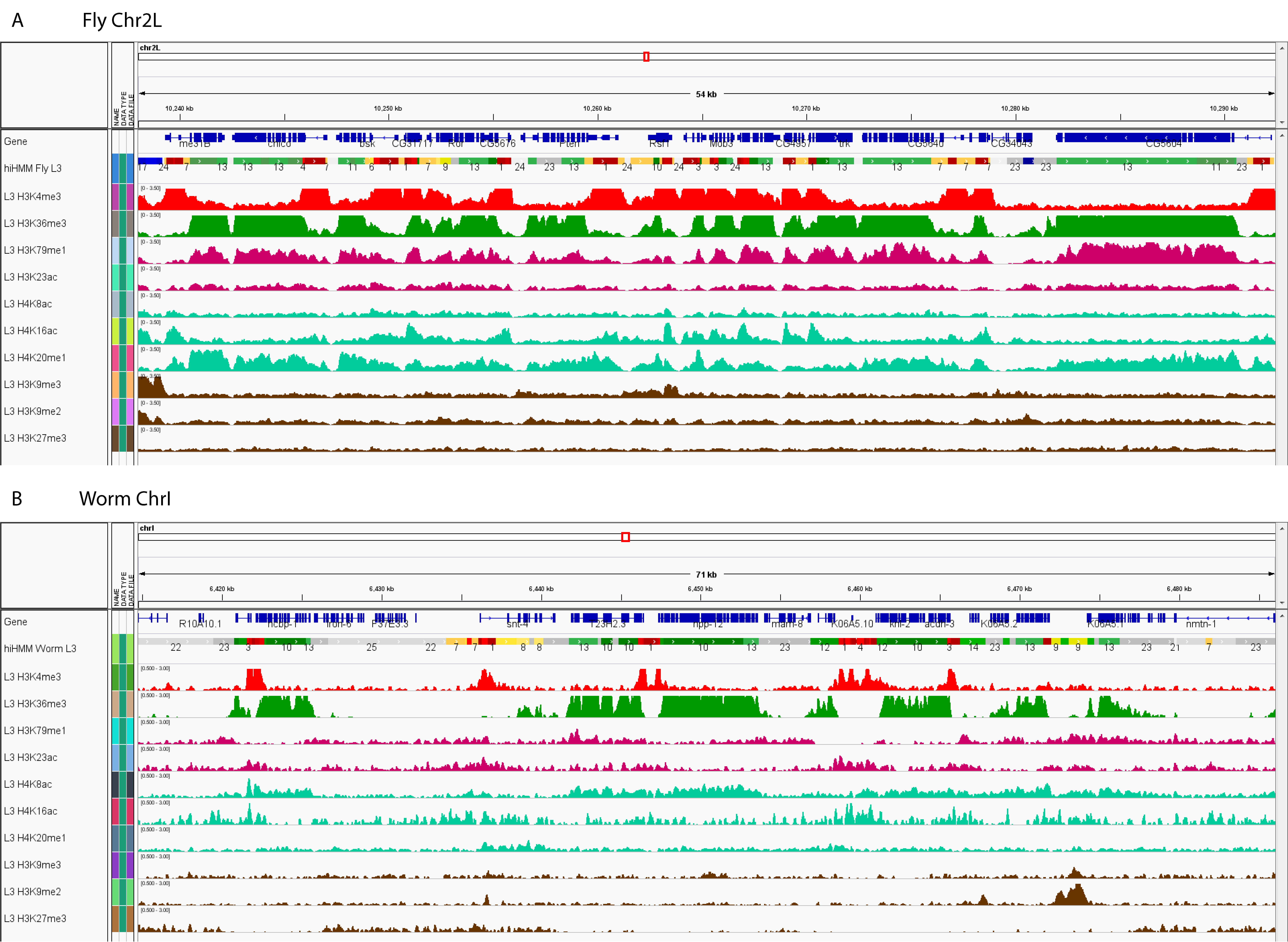
**

**Fig S6. *IGV browser plots showing differences in transcription state composition between fly and worm.*** H4K8ac, H4K16ac and H4K20me1 are coloured in light blue. Transcription states are coloured green in the hiHMM chromatin state track (below gene annotations) and marked by H3K36me3 (green signal).


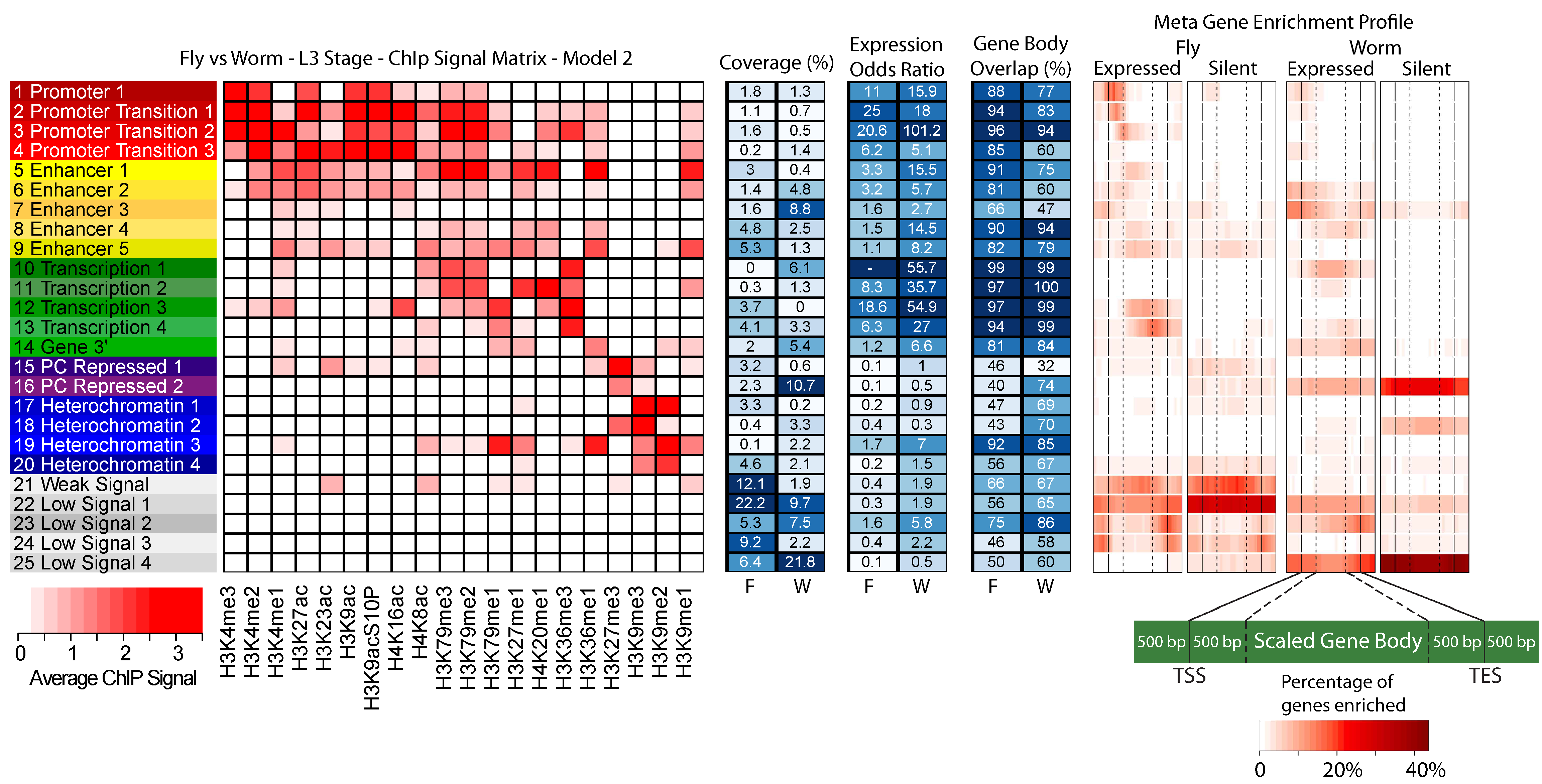


**Fig S7. *Chromatin State Characterisation - Fly vs. Worm - Model 2 (25 States).*** The complete chromatin state characterisation for fly and worm using hiHMM Model 2. Left panels show the average ChIP-seq signal for 20 profiled marks in each state in each species. Right panels show state summary statistics as well as meta gene spatial enrichment profiles.


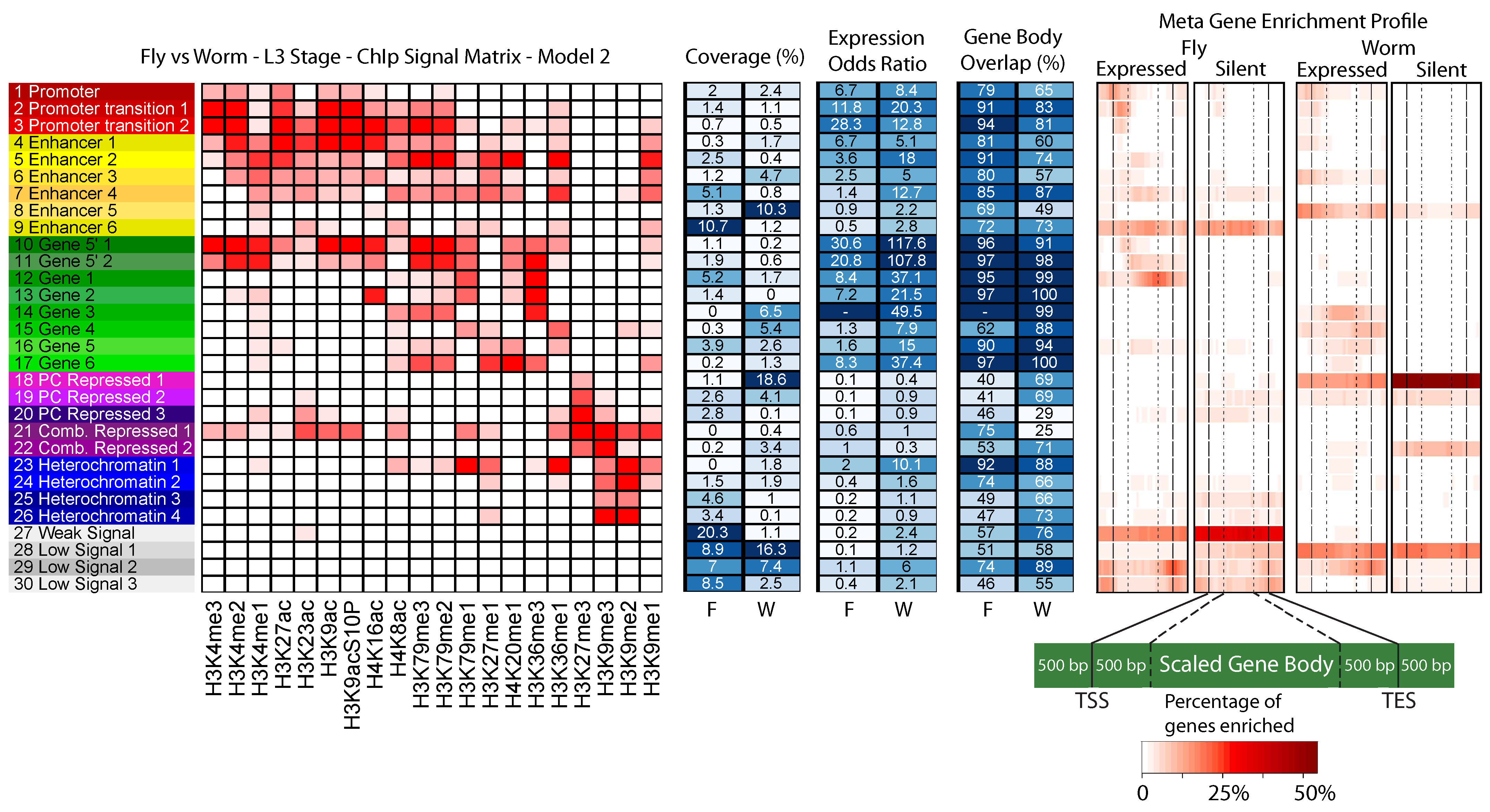


**Fig S8. *Chromatin State Characterisation - Fly vs. Worm - Model 2 (30 States).*** The complete chromatin state characterisation for fly and worm using hiHMM Model 2. Left panels show the average ChIP-seq signal for 20 profiled marks in each state in each species. Right panels show state summary statistics as well as meta gene spatial enrichment profiles.


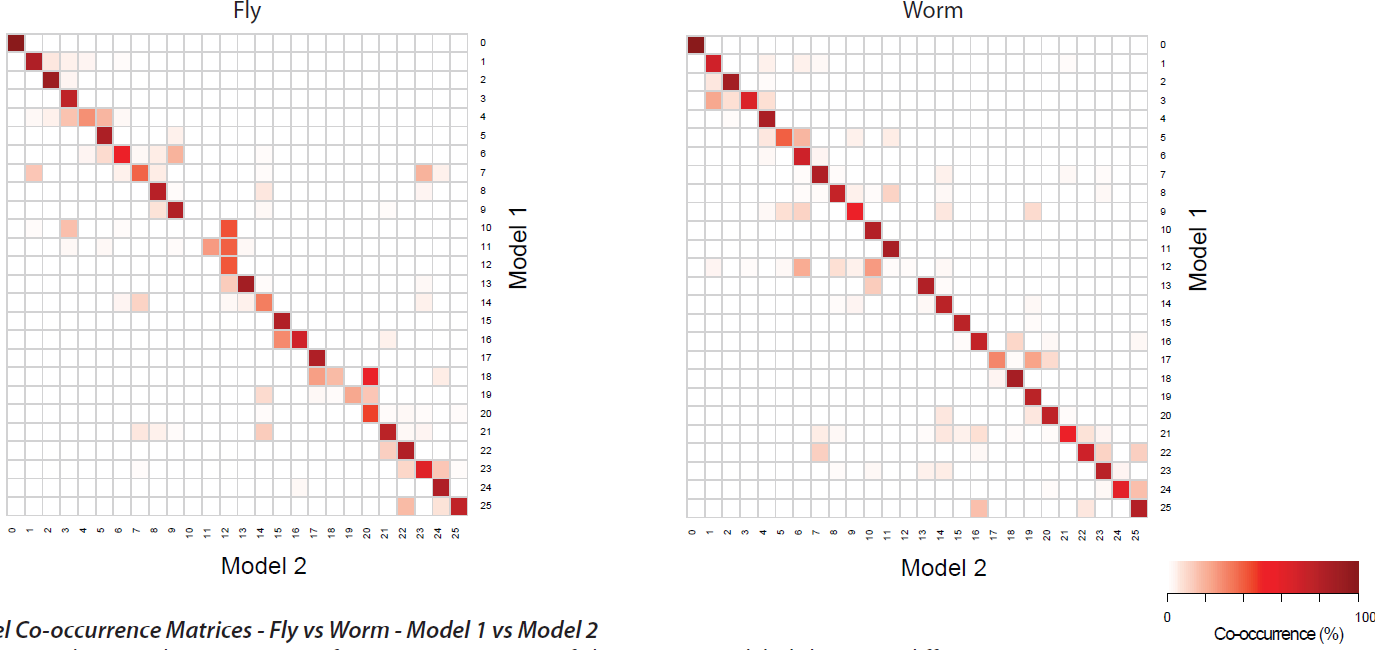


**Fig S9. *Inter-Model Co-occurrence Matrices - Fly vs. Worm - Model 1 vs. Model 2.*** Co-occurrence matrices showing the consistency of genomic annotation of chromatin state labels between different experiments.

*
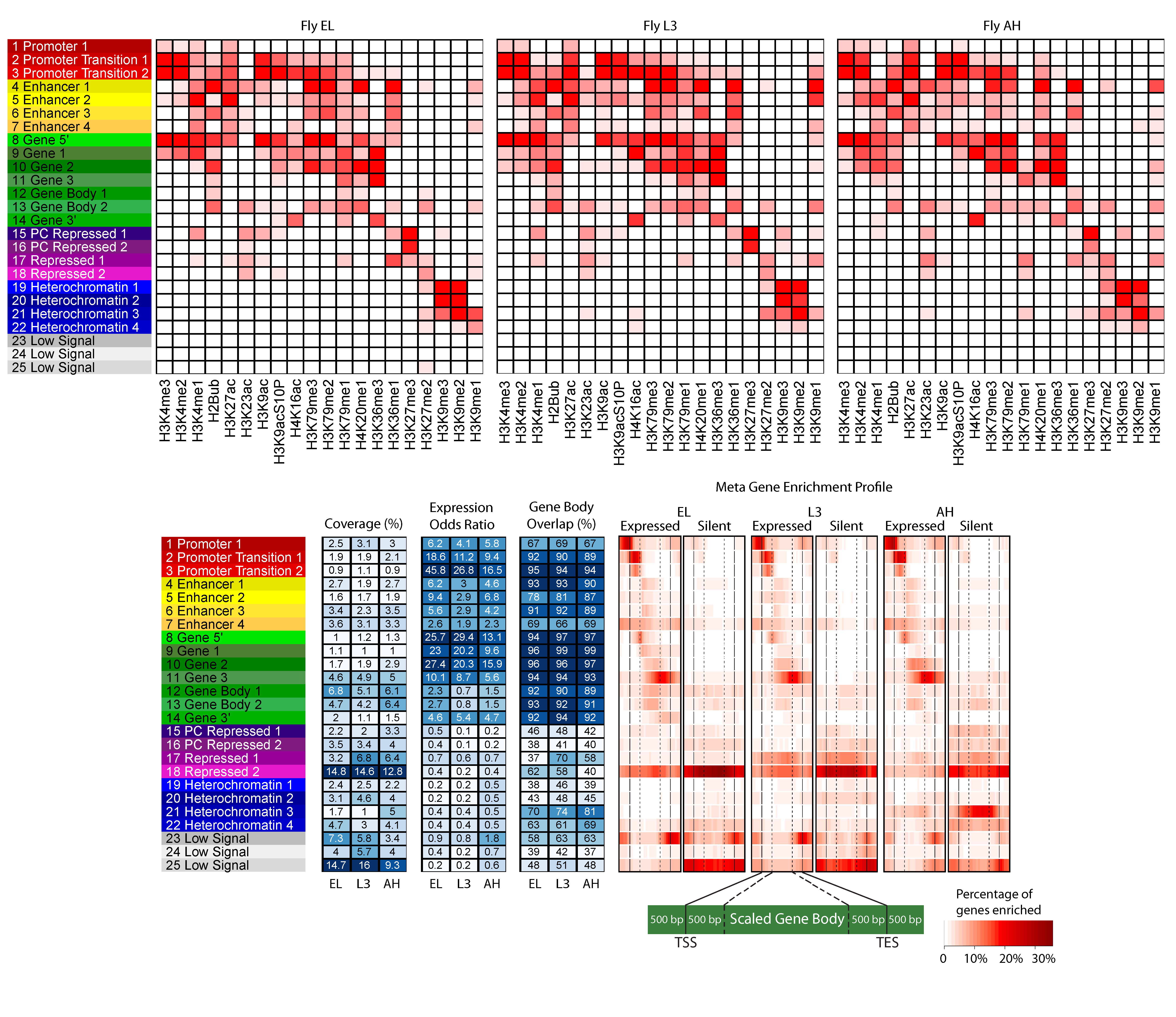
*

**Fig S10. *Chromatin State Characterisation - Fly 3 Stages - Model 1.*** The complete chromatin state characterisation for 3 developmental stages in fly using hiHMM Model 1. Top panels show the average ChIP-Seq signal for 20 profiled marks in each state and at each developmental stage. Bottom panels show state summary statistics as well as meta gene spatial enrichment profiles.


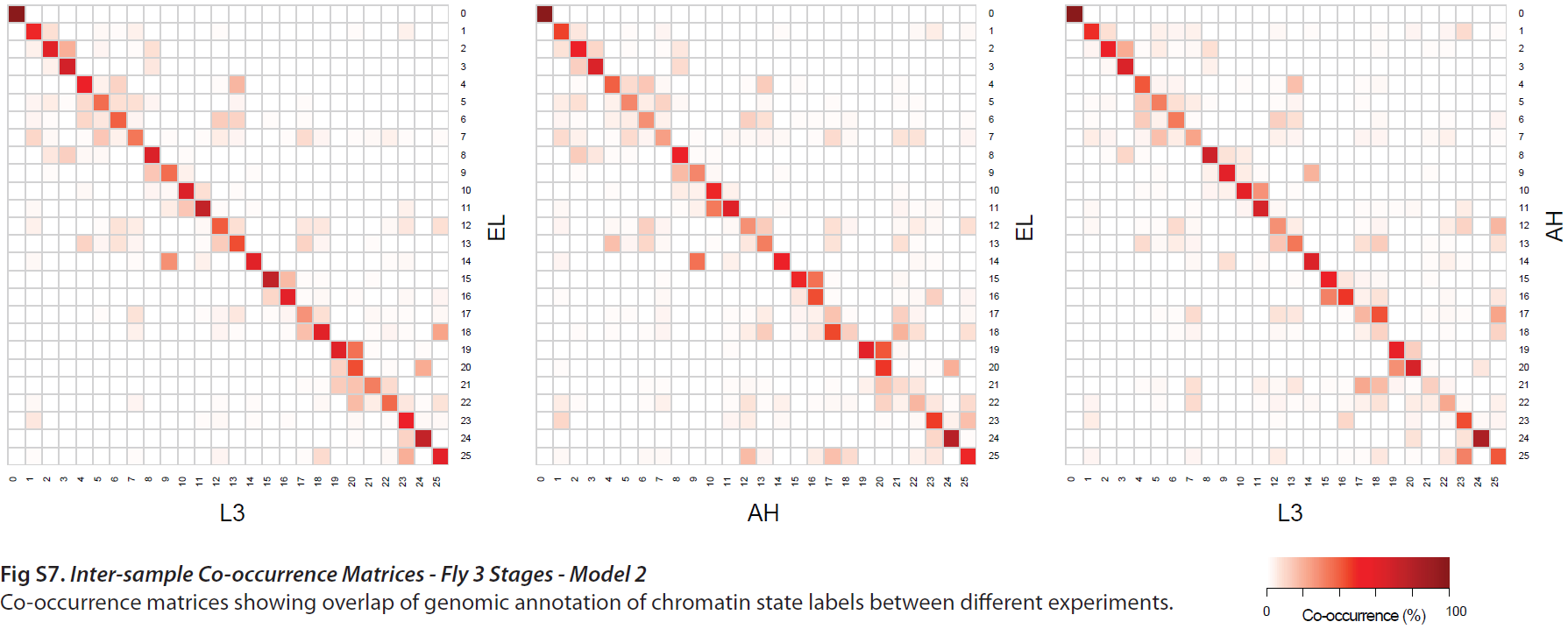


**Fig S11. *Inter-sample Co-occurrence Matrices - Fly 3 Stages - Model 2.*** Co-occurrence matrices showing overlap of genomic annotation of chromatin state labels between different experiments.

**
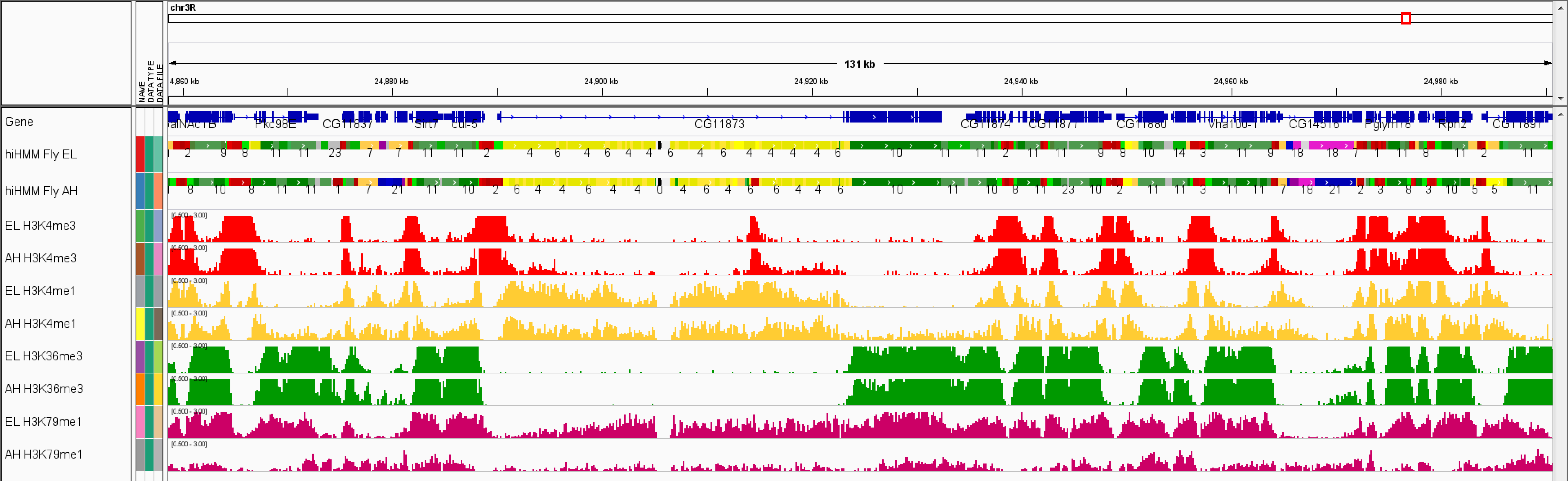
**

**Fig S12. *IGV browser plot showing differences in active state composition between developmental stages in fly.*** For each histone modification two fly developmental stages are shown, EL (above) and AH (below). H3K79me1 is coloured in purple, last two tracks. Active states are coloured red, yellow and green in the hiHMM chromatin state track (below gene annotations) and are marked by H3K4me3 (red signal), H3K4me1 (yellow signal) and H3K36me3 (green signal).


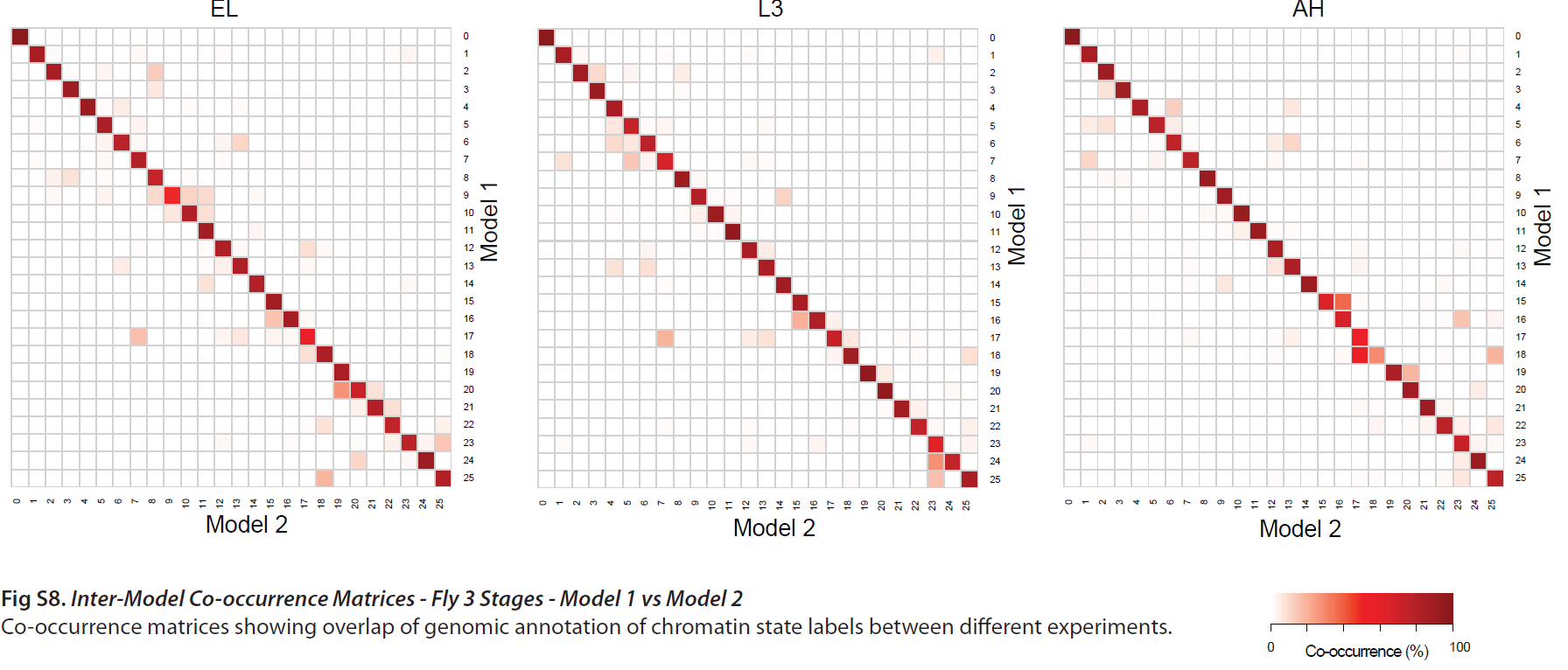


**Fig S13. *Inter-Model Co-occurrence Matrices - Fly 3 Stages - Model 1 vs. Model 2.*** Co-occurrence matrices showing overlap of genomic annotation of chromatin state labels between different experiments.


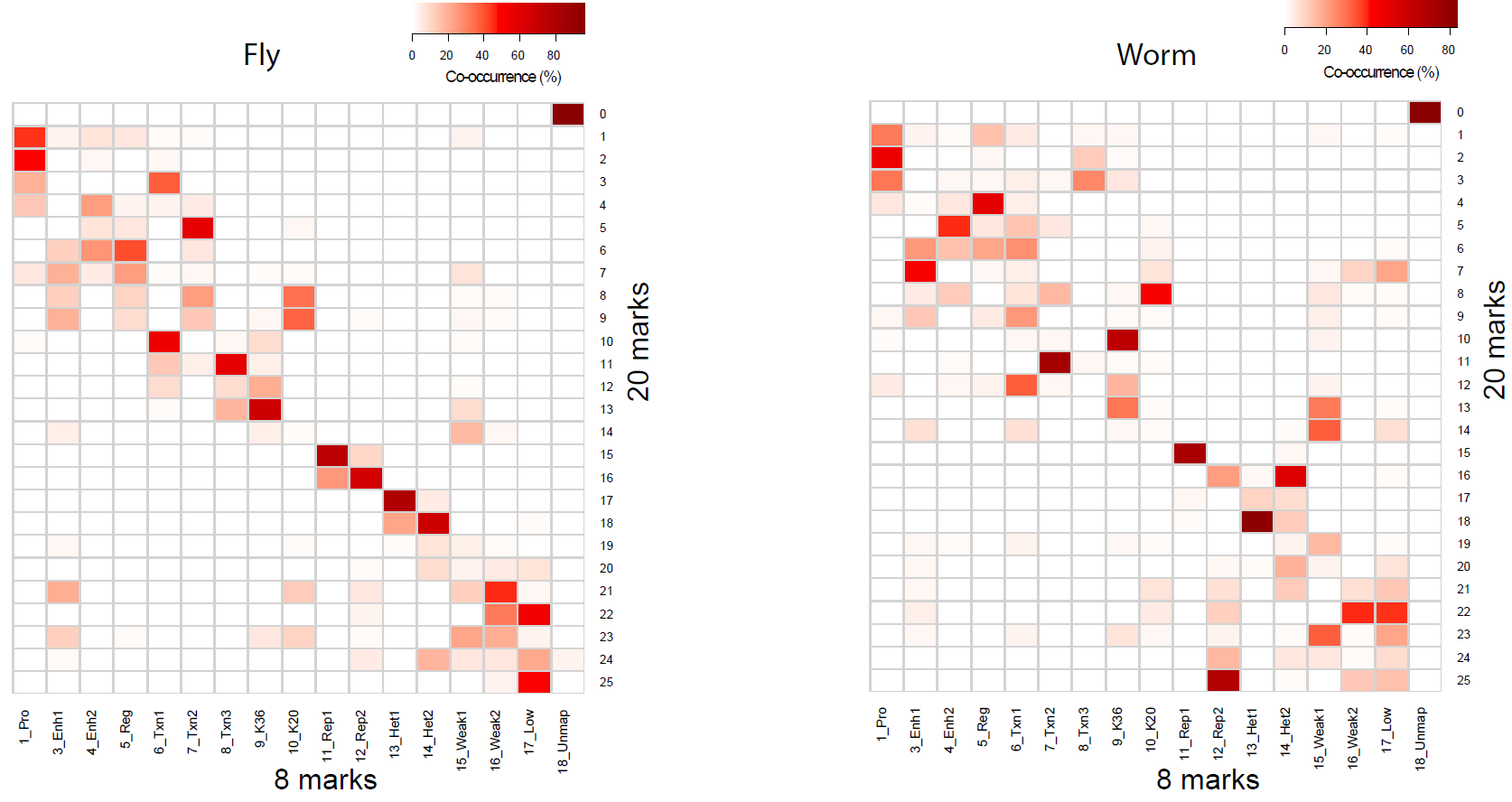


**Fig S14. *Inter-study Co-occurrence matrices.*** Co-occurrence matrices showing the overlap between the 25 states generated by this study using 20 histone modification marks and the 17 state model generated by Ho et al. 2014 using a subset of 8 histone modification marks.

**Fig S15. *Sensitivity to hyper-parameter setting* *(Model 1).*** We consider the effect on the performance of the following three parameters: 1) K_0_, the initial number of states, 2) σ^2^, the variance of the Gaussian that generates observations given a hidden state and the emission matrix, and 3) σ_0_^2^, the variance in the Gaussian for sampling each row of the emission matrix.

*A*. The segmentation accuracy of *F*-measure (left) and adjusted Rand Index (middle). We can see that that the segmentation accuracy is mostly dependent on σ^2^ (the performance at σ^2^=1 is always the best), but less affected by K_0_ or even less by σ_0_^2^ although too small K_0_ seems to degrade the clustering performance by the state labels. This makes sense because the variance in the emission model should be small enough to assign the observation signal to the correct state.

*B*. The number of inferred states is found to depend on all the three parameters, but most significantly on σ_0_^2^. We find that σ_0_^2^ should be large enough to compactly capture the varying signals from enriched marks as the prior mean for the emission matrix is zero. For large σ_0_^2^ (greater than 2), the number of inferred states becomes less affected by other parameters.

*C*. For further investimation, we display the ratio of the inferred number of states to the initial number of states as a function of each parameter. The deviation from the initial number of states is most strongly affected by σ_0_^2^, but not by K_0_ or σ^2^. When σ_0_^2^ gets large, the number of states does not change much through iterations. In this case, the inference algorithm seems to prefer to optimize the posterior by adapting other parameters or variables rather than changing the number of states. It would seem a good practice to set σ_0_^2^ to be around the mean signal strength of the enriched marks, but can be adapted by tracing the number of states through iterations.

**Fig S16. *Sensitivity to hyper-parameter setting* *(Model 2)*.** The same as in Fig S10, but with Model 2. We consider the effect on the performance of the following three parameters: 1) K_0_, the initial number of states, 2) σ^2^, the variance of the Gaussian that generates observations given a hidden state and the emission matrix, and 3) σ_0_^2^, the variance in the Gaussian for sampling each row of the emission matrix. *A*. The segmentation accuracy of *F*-measure (left) and adjusted Rand Index (middle). *B*. The number of inferred states. *C*. The ratio of the inferred number of states to the initial number of states as a function of each parameter.

**Table S1. *Common histone modification marks profiled between samples.*** Different stages of organism development were compared in this study: late embryo (EL); third instar larvae (L3); adult head (AH).


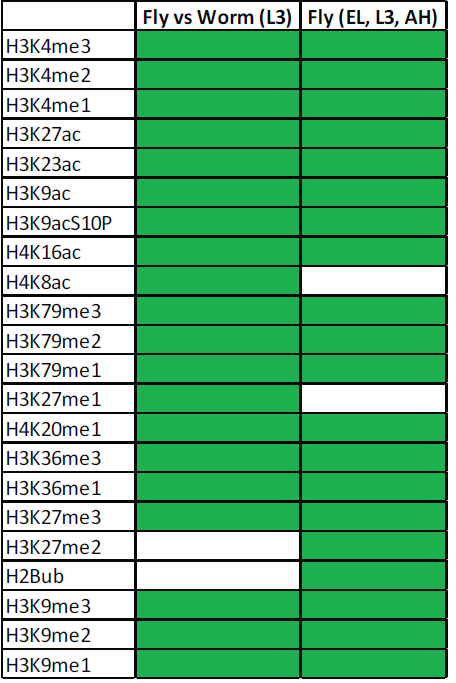

Supplement: Supplementary Data [file supp_btv117_Supplementary_2015_01_26.docx]
